# *catena*-Poly[[[aqua­(2,2′-bipyridine-κ^2^ *N*,*N*′)copper(II)]-μ-furan-2,5-di­carboxyl­ato-κ^2^ *O* ^2^:*O* ^5^] dihydrate]

**DOI:** 10.1107/S1600536812043632

**Published:** 2012-10-27

**Authors:** Ya-Feng Li, Yue Xu, Xiao-Lin Qin, Yong-Peng Yuan, Wen-Yuan Gao

**Affiliations:** a School of Chemical Engineering, Changchun University of Technology, Changchun 130012, People’s Republic of China

## Abstract

In the crystal structure of the title compound, {[Cu(C_6_H_2_O_5_)(C_10_H_8_N_2_)(H_2_O)]·2H_2_O}_*n*_, an infinite chain parallel to [110] is formed by the linking of Cu(H_2_O)(2,2′-bipyridine) units through a furan-2,5-dicarboxyl­ate bridge. The Cu^II^ atom shows a square-pyramidal geometry, with one furan-2,5-dicarboxyl­ate O atom in the apical position. The dihedral angle between the planes of the furan ring and the bipyridine mol­ecule is 83.88 (7)°. O_water_—H⋯O hydrogen bonds connect adjacent chains, generating a layer motif parallel to (001).

## Related literature

For a related structure, see: Li *et al.* (2012 ▶).
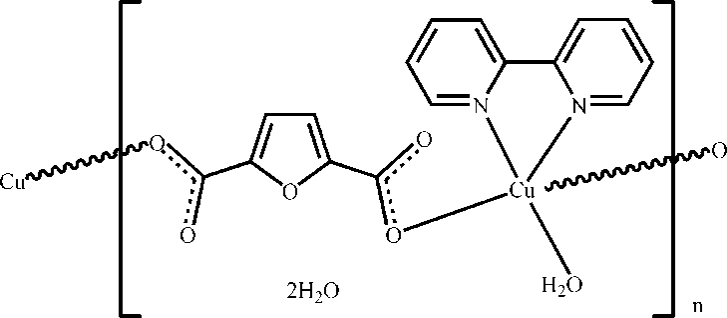


## Experimental

### 

#### Crystal data

[Cu(C_6_H_2_O_5_)(C_10_H_8_N_2_)(H_2_O)]·2H_2_O
*M* *_r_* = 427.86
Triclinic, 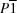

*a* = 8.8621 (18) Å
*b* = 8.9016 (18) Å
*c* = 12.523 (3) Å
α = 88.33 (3)°
β = 69.31 (3)°
γ = 66.85 (3)°
*V* = 842.8 (3) Å^3^
*Z* = 2
Mo *K*α radiation
μ = 1.35 mm^−1^
*T* = 293 K
0.44 × 0.40 × 0.24 mm

#### Data collection

Rigaku R-AXIS RAPID diffractometer
Absorption correction: multi-scan (*ABSCOR*; Higashi, 1995 ▶) *T*_min_ = 0.589, *T*_max_ = 0.738
8312 measured reflections
3809 independent reflections
3430 reflections with *I* > 2σ(*I*)
*R*_int_ = 0.022

#### Refinement

*R*[*F* ^2^ > 2σ(*F* ^2^)] = 0.034
*wR*(*F* ^2^) = 0.092
*S* = 1.09
3809 reflections
262 parameters
8 restraints
H atoms treated by a mixture of independent and constrained refinement
Δρ_max_ = 0.38 e Å^−3^
Δρ_min_ = −0.60 e Å^−3^

### 

Data collection: *PROCESS-AUTO* (Rigaku, 1998 ▶); cell refinement: *PROCESS-AUTO*; data reduction: *CrystalStructure* (Rigaku/MSC, 2002 ▶); program(s) used to solve structure: *SHELXS97* (Sheldrick, 2008 ▶); program(s) used to refine structure: *SHELXL97* (Sheldrick, 2008 ▶); molecular graphics: *DIAMOND* (Brandenburg, 2000 ▶); software used to prepare material for publication: *SHELXL97*.

## Supplementary Material

Crystal structure: contains datablock(s) I, global. DOI: 10.1107/S1600536812043632/ng5302sup1.cif

Structure factors: contains datablock(s) I. DOI: 10.1107/S1600536812043632/ng5302Isup2.hkl

Additional supplementary materials:  crystallographic information; 3D view; checkCIF report

## Figures and Tables

**Table 1:** Hydrogen-bond geometry (Å, °)

*D*—H⋯*A*	*D*—H	H⋯*A*	*D*⋯*A*	*D*—H⋯*A*
O1*W*—H1*A*⋯O1^i^	0.86 (2)	1.87 (2)	2.676 (2)	156 (2)
O1*W*—H1*B*⋯O4^ii^	0.86 (2)	1.78 (2)	2.623 (2)	166 (2)
O2*W*—H2*A*⋯O5	0.86 (2)	2.42 (3)	3.211 (4)	153 (4)
O2*W*—H2*B*⋯O2	0.87 (2)	2.59 (3)	3.353 (4)	146 (4)
O3*W*—H3*A*⋯O4^iii^	0.86 (2)	2.10 (2)	2.891 (3)	153 (4)
O3*W*—H3*B*⋯O2	0.87 (2)	1.96 (2)	2.797 (3)	162 (4)

Symmetry codes: (i) 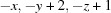; (ii) 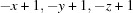; (iii) 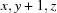.

**Fig. 1.**
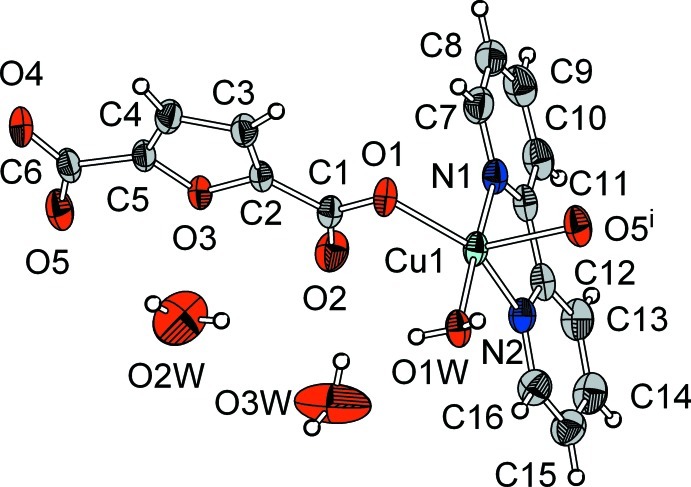
The unit cell of (I), showing the atomic labelling scheme and displacement ellipsoids at the 50% probability level. [Symmetry codes: (i) -1 + x, 1 + y, z.]

**Fig. 2.**
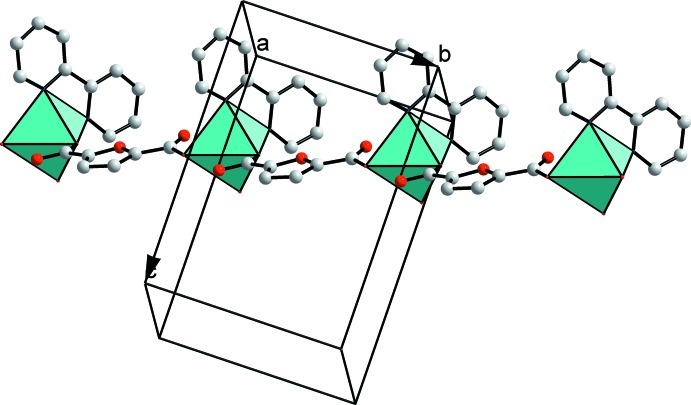
Polyhedral plot of (I), displaying the infinite chain formed by linking the adjacent Cu cations with furan-2,5-dicarboxylate.

**Fig. 3.**
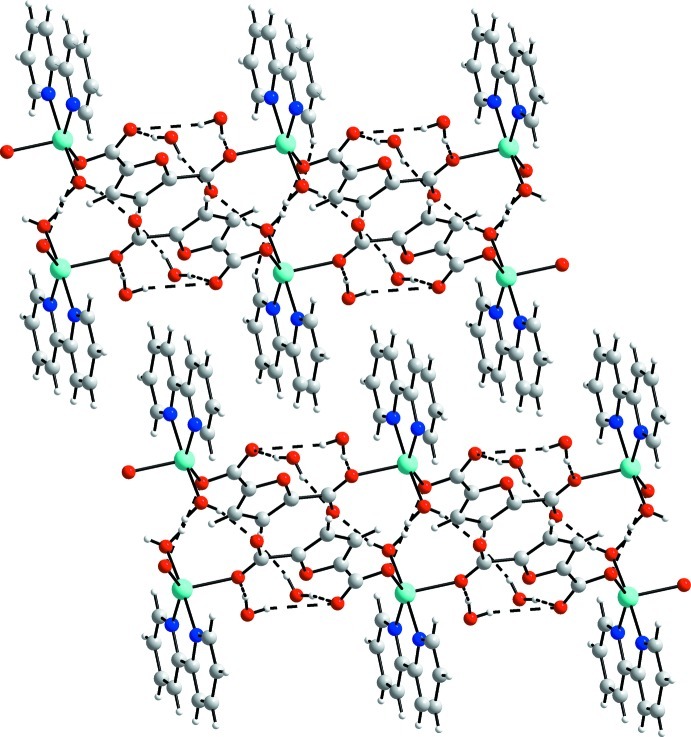
Ball-stick packing diagram of (I). The adjacent chains are holded together by the Owater–H···O H-bonding interactions to the supermolecular net.

## supplementary crystallographic information

### Comment

Recently, we utilized furan-2,5-dicarboxyl acid as the ligand to construct coordination polymers (Li *et al.*, 2012). As an extension of this work, a chainlike compound, [Cu(H_2_O)(C_10_H_8_N_2_)(C_6_H_2_O_5_)]^.^2H_2_O (I), is now determined.

The asymmetric unit of (I) is consisted of one Cu(II) cation, one furan-2,5-dicarboxylate anion, one 2,2'-bipyridine and three water molecule The Cu atom is coordinated by two N atoms of 2,2'-bipyridine, one water O atoms and two carboxylate O atoms, exhibiting distorted square pyramid. Adjacent Cu atoms are connected by the furan-2,5-dicarboxylate to form an infinite chain (Fig. 2); O_water_—H···O hydrogen bonds hold together ajacent chains to form a layer motif (Fig. 3).

### Experimental

Furan-2,5-dicarboxyl acid (0.0156 g, 0.10 mmol), Cu(NO_3_)_2_^.^6H_2_O (0.0298 g, 0.10 mmol), 2,2'-bipyridine (0.0156, 0.10 mmol) and NaOH (0.004, 0.10 mmol) were dissolved in water (5 ml, 278 mmol) under stirring. The mixture with molar ratio of 1 (furan-2,5-dicarboxyl acid): 1 (Cu(NO_3_)_2_^.^6H_2_O): 1 (2,2'-bipyridine): 1 NaOH: 2780 H_2_O was layed under room temperature for 5 days. Blue blocks were collected as a single phase.

### Refinement

Water H atoms were located in a difference Fourier map and refined with O—H = 0.86 (2) Å and *U*_iso_(H) = 1.2Ueq(O). The carbon H-atoms were placed in calculated positions (C—H (furan and pyridine ring) = 0.93 Å) and were included in the refinement in the riding-model approximation, with *U*_iso_(H) = 1.2Ueq(C).

### Crystal data

**Table:**

[Cu(C_6_H_2_O_5_)(C_10_H_8_N_2_)(H_2_O)]·2H_2_O	*Z* = 2
*M**_r_* = 427.86	*F*(000) = 438
Triclinic, *P*1	*D*_x_ = 1.686 Mg m^−^^3^
Hall symbol: -P 1	Mo *K*α radiation, λ = 0.71073 Å
*a* = 8.8621 (18) Å	Cell parameters from 2000 reflections
*b* = 8.9016 (18) Å	θ = 3.2–27.5°
*c* = 12.523 (3) Å	µ = 1.35 mm^−^^1^
α = 88.33 (3)°	*T* = 293 K
β = 69.31 (3)°	Block, blue
γ = 66.85 (3)°	0.44 × 0.40 × 0.24 mm
*V* = 842.8 (3) Å^3^	

### Data collection

**Table:**

Rigaku R-AXIS RAPID diffractometer	3809 independent reflections
Radiation source: fine-focus sealed tube	3430 reflections with *I* > 2σ(*I*)
Graphite monochromator	*R*_int_ = 0.022
Detector resolution: 10.00 pixels mm^-1^	θ_max_ = 27.5°, θ_min_ = 3.2°
ω scans	*h* = −11→11
Absorption correction: multi-scan (*ABSCOR*; Higashi, 1995)	*k* = −10→11
*T*_min_ = 0.589, *T*_max_ = 0.738	*l* = −16→16
8312 measured reflections	

### Refinement

**Table:**

Refinement on *F*^2^	Primary atom site location: structure-invariant direct methods
Least-squares matrix: full	Secondary atom site location: difference Fourier map
*R*[*F*^2^ > 2σ(*F*^2^)] = 0.034	Hydrogen site location: inferred from neighbouring sites
*wR*(*F*^2^) = 0.092	H atoms treated by a mixture of independent and constrained refinement
*S* = 1.09	*w* = 1/[σ^2^(*F*_o_^2^) + (0.0544*P*)^2^ + 0.2206*P*] where *P* = (*F*_o_^2^ + 2*F*_c_^2^)/3
3809 reflections	(Δ/σ)_max_ = 0.001
262 parameters	Δρ_max_ = 0.38 e Å^−^^3^
8 restraints	Δρ_min_ = −0.60 e Å^−^^3^

### Special details

**Table:**

Geometry. All e.s.d.'s (except the e.s.d. in the dihedral angle between two l.s. planes) are estimated using the full covariance matrix. The cell e.s.d.'s are taken into account individually in the estimation of e.s.d.'s in distances, angles and torsion angles; correlations between e.s.d.'s in cell parameters are only used when they are defined by crystal symmetry. An approximate (isotropic) treatment of cell e.s.d.'s is used for estimating e.s.d.'s involving l.s. planes.
Refinement. Refinement of *F*^2^ against ALL reflections. The weighted *R*-factor *wR* and goodness of fit *S* are based on *F*^2^, conventional *R*-factors *R* are based on *F*, with *F* set to zero for negative *F*^2^. The threshold expression of *F*^2^ > σ(*F*^2^) is used only for calculating *R*-factors(gt) *etc*. and is not relevant to the choice of reflections for refinement. *R*-factors based on *F*^2^ are statistically about twice as large as those based on *F*, and *R*- factors based on ALL data will be even larger.

### Fractional atomic coordinates and isotropic or equivalent isotropic displacement parameters (Å^2^)

**Table:**

	*x*	*y*	*z*	*U*_iso_*/*U*_eq_	
Cu1	0.11580 (3)	1.01038 (3)	0.296968 (19)	0.02954 (10)	
O1	0.15473 (18)	0.80049 (16)	0.36822 (12)	0.0331 (3)	
O2	0.4389 (2)	0.7287 (2)	0.26090 (15)	0.0451 (4)	
O3	0.53028 (17)	0.43934 (16)	0.35228 (12)	0.0293 (3)	
O4	0.6968 (2)	0.0548 (2)	0.45196 (17)	0.0512 (4)	
O5	0.8433 (2)	0.19001 (19)	0.33789 (14)	0.0428 (4)	
N1	0.1301 (2)	0.9088 (2)	0.15292 (15)	0.0326 (4)	
N2	0.2242 (2)	1.1413 (2)	0.18474 (14)	0.0312 (3)	
C1	0.3191 (3)	0.7013 (2)	0.33247 (16)	0.0288 (4)	
C2	0.3569 (2)	0.5458 (2)	0.38399 (16)	0.0261 (4)	
C3	0.2525 (3)	0.4845 (2)	0.46097 (19)	0.0335 (4)	
H3	0.1299	0.5339	0.4956	0.040*	
C4	0.3660 (3)	0.3303 (3)	0.4786 (2)	0.0366 (4)	
H4	0.3327	0.2579	0.5265	0.044*	
C5	0.5319 (3)	0.3088 (2)	0.41239 (17)	0.0294 (4)	
C6	0.7064 (3)	0.1739 (2)	0.39860 (18)	0.0338 (4)	
C7	0.0776 (3)	0.7887 (3)	0.1463 (2)	0.0421 (5)	
H7	0.0250	0.7516	0.2136	0.050*	
C8	0.0994 (4)	0.7183 (3)	0.0416 (2)	0.0478 (6)	
H8	0.0612	0.6357	0.0385	0.057*	
C9	0.1790 (3)	0.7732 (3)	−0.0579 (2)	0.0486 (6)	
H9	0.1979	0.7257	−0.1292	0.058*	
C10	0.2306 (3)	0.8992 (3)	−0.05098 (19)	0.0416 (5)	
H10	0.2828	0.9386	−0.1172	0.050*	
C11	0.2033 (3)	0.9656 (2)	0.05623 (17)	0.0320 (4)	
C12	0.2515 (3)	1.1018 (2)	0.07481 (17)	0.0310 (4)	
C13	0.3209 (3)	1.1829 (3)	−0.01247 (19)	0.0409 (5)	
H13	0.3389	1.1543	−0.0882	0.049*	
C14	0.3626 (3)	1.3065 (3)	0.0148 (2)	0.0455 (5)	
H14	0.4086	1.3629	−0.0424	0.055*	
C15	0.3359 (3)	1.3460 (3)	0.1276 (2)	0.0439 (5)	
H15	0.3648	1.4284	0.1472	0.053*	
C16	0.2659 (3)	1.2617 (3)	0.2105 (2)	0.0398 (5)	
H16	0.2468	1.2889	0.2867	0.048*	
O1W	0.1177 (2)	1.12059 (17)	0.43017 (12)	0.0325 (3)	
H1A	0.014 (2)	1.164 (3)	0.4834 (19)	0.039*	
H1B	0.191 (3)	1.056 (3)	0.458 (2)	0.039*	
O2W	0.8652 (4)	0.5101 (4)	0.2205 (3)	0.0910 (9)	
H2A	0.832 (6)	0.451 (5)	0.272 (3)	0.109*	
H2B	0.767 (4)	0.599 (4)	0.241 (4)	0.109*	
O3W	0.6105 (5)	0.9237 (5)	0.2840 (2)	0.1073 (12)	
H3A	0.611 (7)	0.952 (6)	0.349 (3)	0.129*	
H3B	0.540 (6)	0.876 (6)	0.291 (4)	0.129*	

### Atomic displacement parameters (Å^2^)

**Table:**

	*U*^11^	*U*^22^	*U*^33^	*U*^12^	*U*^13^	*U*^23^
Cu1	0.03370 (15)	0.02750 (14)	0.02279 (14)	−0.00666 (10)	−0.01233 (10)	0.00782 (9)
O1	0.0304 (7)	0.0281 (6)	0.0285 (7)	−0.0017 (5)	−0.0093 (6)	0.0085 (5)
O2	0.0393 (9)	0.0437 (9)	0.0430 (9)	−0.0158 (7)	−0.0071 (7)	0.0168 (7)
O3	0.0238 (6)	0.0268 (6)	0.0306 (7)	−0.0038 (5)	−0.0104 (5)	0.0055 (5)
O4	0.0553 (11)	0.0374 (8)	0.0717 (12)	−0.0114 (7)	−0.0454 (10)	0.0227 (8)
O5	0.0294 (8)	0.0399 (8)	0.0448 (9)	0.0019 (6)	−0.0154 (7)	0.0012 (6)
N1	0.0358 (9)	0.0294 (8)	0.0298 (9)	−0.0069 (7)	−0.0167 (7)	0.0074 (6)
N2	0.0314 (8)	0.0318 (8)	0.0245 (8)	−0.0078 (7)	−0.0098 (7)	0.0066 (6)
C1	0.0322 (10)	0.0252 (8)	0.0258 (9)	−0.0070 (7)	−0.0127 (8)	0.0042 (7)
C2	0.0230 (8)	0.0244 (8)	0.0271 (9)	−0.0037 (6)	−0.0116 (7)	0.0025 (7)
C3	0.0257 (9)	0.0316 (9)	0.0396 (11)	−0.0084 (7)	−0.0121 (8)	0.0099 (8)
C4	0.0370 (11)	0.0322 (10)	0.0431 (12)	−0.0133 (8)	−0.0192 (9)	0.0160 (8)
C5	0.0314 (10)	0.0254 (8)	0.0318 (10)	−0.0063 (7)	−0.0184 (8)	0.0058 (7)
C6	0.0356 (11)	0.0290 (9)	0.0349 (11)	−0.0025 (8)	−0.0228 (9)	−0.0001 (7)
C7	0.0499 (13)	0.0355 (11)	0.0435 (13)	−0.0141 (9)	−0.0246 (11)	0.0110 (9)
C8	0.0538 (14)	0.0353 (11)	0.0593 (16)	−0.0112 (10)	−0.0343 (13)	0.0032 (10)
C9	0.0499 (14)	0.0471 (13)	0.0412 (13)	−0.0049 (10)	−0.0247 (11)	−0.0062 (10)
C10	0.0378 (11)	0.0467 (12)	0.0291 (11)	−0.0050 (9)	−0.0134 (9)	0.0008 (9)
C11	0.0265 (9)	0.0326 (9)	0.0278 (10)	−0.0014 (7)	−0.0123 (8)	0.0040 (7)
C12	0.0255 (9)	0.0337 (9)	0.0246 (9)	−0.0028 (7)	−0.0096 (8)	0.0056 (7)
C13	0.0409 (12)	0.0465 (12)	0.0253 (10)	−0.0117 (9)	−0.0086 (9)	0.0099 (8)
C14	0.0428 (13)	0.0471 (12)	0.0382 (12)	−0.0167 (10)	−0.0082 (10)	0.0157 (10)
C15	0.0453 (13)	0.0410 (12)	0.0443 (13)	−0.0194 (10)	−0.0141 (11)	0.0111 (9)
C16	0.0459 (12)	0.0402 (11)	0.0329 (11)	−0.0168 (9)	−0.0154 (10)	0.0074 (8)
O1W	0.0319 (7)	0.0338 (7)	0.0247 (7)	−0.0049 (6)	−0.0125 (6)	0.0064 (5)
O2W	0.0754 (18)	0.0805 (18)	0.091 (2)	−0.0308 (14)	−0.0024 (16)	0.0061 (15)
O3W	0.166 (3)	0.179 (3)	0.0414 (12)	−0.140 (3)	−0.0327 (16)	0.0241 (16)

### Geometric parameters (Å, º)

**Table:**

Cu1—O1W	1.9681 (15)	C7—H7	0.9300
Cu1—N1	1.9825 (18)	C8—C9	1.380 (4)
Cu1—O1	2.0048 (15)	C8—H8	0.9300
Cu1—N2	2.0218 (18)	C9—C10	1.382 (4)
Cu1—O5^i^	2.1885 (18)	C9—H9	0.9300
O1—C1	1.286 (2)	C10—C11	1.384 (3)
O2—C1	1.228 (3)	C10—H10	0.9300
O3—C5	1.364 (2)	C11—C12	1.483 (3)
O3—C2	1.364 (2)	C12—C13	1.386 (3)
O4—C6	1.252 (3)	C13—C14	1.377 (4)
O5—C6	1.244 (3)	C13—H13	0.9300
N1—C7	1.338 (3)	C14—C15	1.378 (4)
N1—C11	1.345 (3)	C14—H14	0.9300
N2—C12	1.343 (3)	C15—C16	1.376 (3)
N2—C16	1.343 (3)	C15—H15	0.9300
C1—C2	1.477 (2)	C16—H16	0.9300
C2—C3	1.349 (3)	O1W—H1A	0.861 (15)
C3—C4	1.413 (3)	O1W—H1B	0.856 (16)
C3—H3	0.9300	O2W—H2A	0.862 (19)
C4—C5	1.346 (3)	O2W—H2B	0.872 (19)
C4—H4	0.9300	O3W—H3A	0.862 (18)
C5—C6	1.493 (3)	O3W—H3B	0.868 (18)
C7—C8	1.386 (3)		
			
O1W—Cu1—N1	174.26 (7)	O5—C6—C5	118.49 (19)
O1W—Cu1—O1	91.08 (6)	O5—C6—C5	118.49 (19)
N1—Cu1—O1	92.85 (7)	O4—C6—C5	114.5 (2)
O1W—Cu1—N2	93.35 (7)	N1—C7—C8	121.7 (2)
N1—Cu1—N2	81.09 (7)	N1—C7—H7	119.1
O1—Cu1—N2	147.26 (7)	C8—C7—H7	119.1
O1W—Cu1—O5^i^	88.22 (7)	C9—C8—C7	118.8 (2)
N1—Cu1—O5^i^	93.61 (8)	C9—C8—H8	120.6
O1—Cu1—O5^i^	117.97 (7)	C7—C8—H8	120.6
N2—Cu1—O5^i^	94.59 (7)	C8—C9—C10	119.6 (2)
C1—O1—Cu1	112.08 (13)	C8—C9—H9	120.2
C5—O3—C2	106.11 (15)	C10—C9—H9	120.2
C6—O5—Cu1^ii^	126.72 (14)	C9—C10—C11	118.9 (2)
C7—N1—C11	119.63 (18)	C9—C10—H10	120.6
C7—N1—Cu1	124.99 (15)	C11—C10—H10	120.6
C11—N1—Cu1	115.35 (14)	N1—C11—C10	121.4 (2)
C12—N2—C16	119.17 (18)	N1—C11—C12	114.70 (17)
C12—N2—Cu1	114.27 (14)	C10—C11—C12	123.9 (2)
C16—N2—Cu1	126.55 (14)	N2—C12—C13	121.52 (19)
O2—C1—O1	124.51 (18)	N2—C12—C11	114.49 (18)
O2—C1—O1	124.51 (18)	C13—C12—C11	124.00 (18)
O2—C1—C2	120.80 (18)	C14—C13—C12	118.9 (2)
O2—C1—C2	120.80 (18)	C14—C13—H13	120.6
O1—C1—C2	114.69 (17)	C12—C13—H13	120.6
C3—C2—O3	110.33 (16)	C13—C14—C15	119.6 (2)
C3—C2—C1	132.79 (17)	C13—C14—H14	120.2
O3—C2—C1	116.87 (17)	C15—C14—H14	120.2
C2—C3—C4	106.52 (18)	C16—C15—C14	118.9 (2)
C2—C3—H3	126.7	C16—C15—H15	120.6
C4—C3—H3	126.7	C14—C15—H15	120.6
C5—C4—C3	106.56 (19)	N2—C16—C15	122.0 (2)
C5—C4—H4	126.7	N2—C16—H16	119.0
C3—C4—H4	126.7	C15—C16—H16	119.0
C4—C5—O3	110.48 (17)	Cu1—O1W—H1A	111.7 (16)
C4—C5—C6	131.21 (19)	Cu1—O1W—H1B	112.7 (16)
O3—C5—C6	118.28 (18)	H1A—O1W—H1B	109.8 (19)
O5—C6—O4	127.0 (2)	H2A—O2W—H2B	100 (4)
O5—C6—O4	127.0 (2)	H3A—O3W—H3B	113 (3)
			
O1W—Cu1—O1—C1	−95.25 (14)	Cu1^ii^—O5—C6—O5	0 (100)
N1—Cu1—O1—C1	80.57 (14)	O5—O5—C6—O4	0.00 (8)
N2—Cu1—O1—C1	2.6 (2)	Cu1^ii^—O5—C6—O4	2.9 (3)
O5^i^—Cu1—O1—C1	176.19 (12)	O5—O5—C6—C5	0.00 (6)
O1—Cu1—N1—C7	32.30 (18)	Cu1^ii^—O5—C6—C5	−177.61 (12)
N2—Cu1—N1—C7	179.94 (18)	C4—C5—C6—O5	−174.2 (2)
O5^i^—Cu1—N1—C7	−85.97 (18)	O3—C5—C6—O5	3.8 (3)
O1—Cu1—N1—C11	−145.90 (14)	C4—C5—C6—O5	−174.2 (2)
N2—Cu1—N1—C11	1.74 (14)	O3—C5—C6—O5	3.8 (3)
O5^i^—Cu1—N1—C11	95.83 (15)	C4—C5—C6—O4	5.4 (3)
O1W—Cu1—N2—C12	178.94 (14)	O3—C5—C6—O4	−176.59 (17)
N1—Cu1—N2—C12	0.37 (14)	C11—N1—C7—C8	1.2 (3)
O1—Cu1—N2—C12	81.71 (18)	Cu1—N1—C7—C8	−176.89 (17)
O5^i^—Cu1—N2—C12	−92.58 (14)	N1—C7—C8—C9	0.6 (4)
O1W—Cu1—N2—C16	−2.47 (18)	C7—C8—C9—C10	−1.7 (4)
N1—Cu1—N2—C16	178.95 (19)	C8—C9—C10—C11	1.0 (3)
O1—Cu1—N2—C16	−99.7 (2)	C7—N1—C11—C10	−2.0 (3)
O5^i^—Cu1—N2—C16	86.00 (18)	Cu1—N1—C11—C10	176.33 (15)
O2—O2—C1—O1	0.00 (8)	C7—N1—C11—C12	178.33 (18)
O2—O2—C1—C2	0.00 (14)	Cu1—N1—C11—C12	−3.4 (2)
Cu1—O1—C1—O2	−1.8 (3)	C9—C10—C11—N1	0.9 (3)
Cu1—O1—C1—O2	−1.8 (3)	C9—C10—C11—C12	−179.47 (19)
Cu1—O1—C1—C2	178.61 (12)	C16—N2—C12—C13	−0.3 (3)
C5—O3—C2—C3	−0.3 (2)	Cu1—N2—C12—C13	178.40 (16)
C5—O3—C2—C1	178.50 (15)	C16—N2—C12—C11	179.10 (17)
O2—C1—C2—C3	−178.6 (2)	Cu1—N2—C12—C11	−2.2 (2)
O2—C1—C2—C3	−178.6 (2)	N1—C11—C12—N2	3.7 (2)
O1—C1—C2—C3	1.1 (3)	C10—C11—C12—N2	−176.01 (19)
O2—C1—C2—O3	2.9 (3)	N1—C11—C12—C13	−176.94 (19)
O2—C1—C2—O3	2.9 (3)	C10—C11—C12—C13	3.4 (3)
O1—C1—C2—O3	−177.42 (16)	N2—C12—C13—C14	0.1 (3)
O3—C2—C3—C4	−0.2 (2)	C11—C12—C13—C14	−179.3 (2)
C1—C2—C3—C4	−178.8 (2)	C12—C13—C14—C15	0.5 (3)
C2—C3—C4—C5	0.7 (2)	C13—C14—C15—C16	−0.8 (4)
C3—C4—C5—O3	−0.9 (2)	C12—N2—C16—C15	0.0 (3)
C3—C4—C5—C6	177.18 (19)	Cu1—N2—C16—C15	−178.54 (18)
C2—O3—C5—C4	0.8 (2)	C14—C15—C16—N2	0.5 (4)
C2—O3—C5—C6	−177.60 (15)		

Symmetry codes: (i) *x*−1, *y*+1, *z*; (ii) *x*+1, *y*−1, *z*.

### Hydrogen-bond geometry (Å, º)

**Table:**

*D*—H···*A*	*D*—H	H···*A*	*D*···*A*	*D*—H···*A*
O1*W*—H1*A*···O1^iii^	0.86 (2)	1.87 (2)	2.676 (2)	156 (2)
O1*W*—H1*B*···O4^iv^	0.86 (2)	1.78 (2)	2.623 (2)	166 (2)
O2*W*—H2*A*···O5	0.86 (2)	2.42 (3)	3.211 (4)	153 (4)
O2*W*—H2*B*···O2	0.87 (2)	2.59 (3)	3.353 (4)	146 (4)
O3*W*—H3*A*···O4^v^	0.86 (2)	2.10 (2)	2.891 (3)	153 (4)
O3*W*—H3*B*···O2	0.87 (2)	1.96 (2)	2.797 (3)	162 (4)

Symmetry codes: (iii) −*x*, −*y*+2, −*z*+1; (iv) −*x*+1, −*y*+1, −*z*+1; (v) *x*, *y*+1, *z*.

## References

[bb1] Brandenburg, K. (2000). *DIAMOND* Crystal Impact GbR, Bonn, Germany.

[bb2] Higashi, T. (1995). *ABSCOR* Rigaku Corporation, Tokyo, Japan.

[bb3] Li, Y.-F., Xu, Y., Qin, X.-L., Yuan, Y.-P. & Gao, W.-Y. (2012). *Acta Cryst.* E**68**, m1140. 10.1107/S160053681203111X PMC3435570 22969443

[bb4] Rigaku (1998). *PROCESS-AUTO* Rigaku Corporation, Tokyo, Japan.

[bb5] Rigaku/MSC (2002). *CrystalStructure* Rigaku/MSC Inc., The Woodlands, Texas, USA.

[bb6] Sheldrick, G. M. (2008). *Acta Cryst.* A**64**, 112–122. 10.1107/S0108767307043930 18156677

